# Calcified right atrial thrombus in HIV infected patient

**DOI:** 10.11604/pamj.2013.14.166.2176

**Published:** 2013-04-30

**Authors:** Julius Chacha Mwita, Monkgogi Goepamang, Jack Joseph Mkubwa, Teeluck Kumar Gunness, Deshmukh Reebye, Kelebogile Motumise

**Affiliations:** 1 Department of internal medicine, University of Botswana, Botswana; 2 Princess Marina Hospital, Botswana; 3 Cardiac Centre, Pamplemousses, Mauritius

**Keywords:** Calcified right atrial thrombus, pulmonary embolism, Botswana

## Abstract

Calcified right atrial thrombi are rare cardiac masses that may be complicated by pulmonary embolism. Although they can be discovered by a transthoracic echocardiography, they may need histological examination to differentiate them from other cardiac masses. We report a case of a 44-year-old woman who presented with a calcified right atrial thrombus and progressive dyspnoea.

## Introduction

Calcified right sided masses may results from neoplastic or non-neoplastic processes [1].Their differential diagnoses include calcified mural thrombus, cardiac fibroma, osteosarcoma, vegetation and cysts [2]. However, preoperative diagnosis of calcified intra-cardiac masses may be challenging.

## Patient and observation

A 44-year-old woman with HIV/AIDS of 8 years duration developed progressive breathlessness on exertion and fatigue over the past two years. She denied any cough, chest pain, paroxysmal nocturnal dyspnoea, or orthopnoea. Her past medical history was significant of well controlled diabetes mellitus and hypertension.

It was learnt that the patient had been followed-up for a right atrial thrombus for 2 years and had received warfarin since then. She has also been on Combivir (zidovudine + lamivudine) and nevirapine over 8 years. Her most recent CD4 count was 367 units/ml with undetectable viral loads. In 2004, she presented with renal insufficient and nephrotic range proteinuria that subsequently disappeared after a short course of oral steroids and initiation of highly active antiretrovirals (HAART). She has, since then, been followed for a stable stage 3 chronic kidney disease. She stopped cigarette smoking and alcohol drinking about two years ago. She denied any history of drug abuse and she had never been on oral contraceptive pills. On admission, she was clinically afebrile but desaturating at SO_2_ 85% on room air. Her blood pressure was 117/76 mm Hg and she had a pulse rate of 96 beats per minute. There was a marked dilatation of both jugular veins and mild bilateral ankle oedema. She had no murmurs and her lungs were clear. A 12-lead electrocardiogram detected sinus rhythm of 96 beats per minutes, right axis deviation, and an incomplete right bundle-branch block. A chest X- ray showed moderate cardiomegaly with clear lung fields. Her laboratory results were: WBC, 6370/ml; Haemoglobin, 13.3g/dl; platelets, 274 × 10^9^/l; Sodium, 140mmol/l; Potassium, 5.5mmol/l; Urea, 13.3mmol/l; Creatinine, 181µmol/l; ALP, 354U/l; ALT, 23.2u/l; AST, 37.3U/l; total protein, 86.5g/l; albumin, 37.3g/l; and glucose 12.88mmol/l. Arterial blood gas showed: PH, 7.42; PC0_2_, 40mmHg; PO_2_, 56mmHg and HC0_3_ 25.1mmol/l.

**Figure 1:**
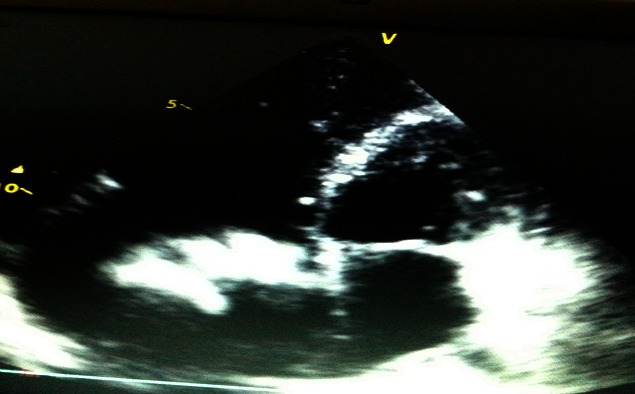
Transthoracic echocardiography showing a right atrial polyploidy mass, right atrial and ventricular dilatation

**Figure 2:**
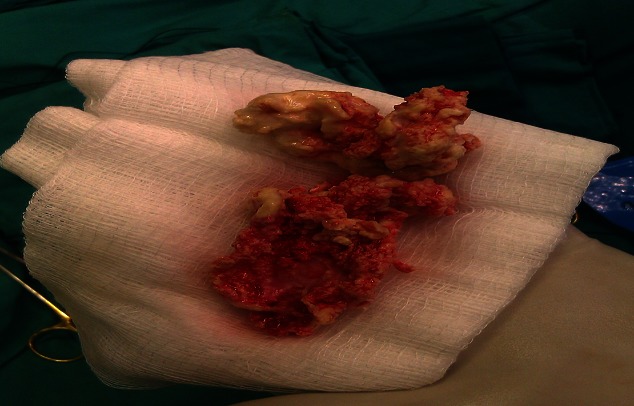
A calcified thrombus that was removed from the right atrium

Two dimensional transthoracic and trans-oesophageal echocardiographs showed a large lobulated and mobile mass in the right atrium (Figure 1). It was attached posteriorly on the lower part of the right atrium and extended into inferior vena cava. The tricuspid valves were not distinctively visible. The mass was moving to and fro into the right ventricle with each cycle without any significant occlusion of the tricuspid valve (Figure 1). There was a free flow of blood between the right atrium and ventricle. The right atrium and ventricle were dilated. The right and left ventricular systolic functions were normal. There was no evidence of pericardial effusion. The superior vena cava was normal. The abdominal ultrasound confirmed the extension of the mass into the inferior vena cava. Other abdominal findings were otherwise unremarkable. Despite over two years of anticoagulant therapy, the mass remained unchanged in size. She consequently underwent surgery. At operation, a rubbery, creamy coloured and mobile atrial mass (5 × 5 × 3 cm) arising from the inferior vena cava (IVC) was appreciated. It was free from the right atrial wall and the tricuspid valve but extended into the inferior vena cava. The atrial mass was easily detached from the junction of the inferior vena cava and removed completely (Figure 2). The extension into the IVC was fixed to the caval walls. It was not removed as this would have ruptured the inferior vena cava. Histology revealed a fibrous tissue with embedded red cells and extensive calcification, compatible with a calcified thrombus. The patient died 24 hours postoperatively due persistent hypotension and progressive right failure. The chest was re-opened before she died and revealed a tense dilated right atrium, hypokinetic right ventricle with no evidence of pericardial effusion. She most likely died from pulmonary embolism caused by fragments from the IVC thrombus.

## Discussion

The differential diagnosis of right atrial mass includes vegetation, tumour, and thrombus. Right atrial thrombi are infrequent and may be associated with hazardous complications including pulmonary embolism [3]. They can develop in situ or as a result of an embolus from systemic vein thrombosis [3]. In situ thrombosis is uncommon in a structurally normal heart. It can nevertheless occur in hypercoagulable states, right atrial catheterization, malignant states, low cardiac output states, cardiomyopathies, cardiac arrhythmias, chronic kidney disease and some systemic diseases [1]. They are usually adherent to the wall of the right atrium and are less likely to embolize. They are associated with good prognosis. In contrast, mobile right atrial thrombi are usually on transit from the deep leg veins to the pulmonary arteries [3]. Since they are often freely floating in the right atrium, they have a high potential to embolize and hence entail emergency treatment [3, 4]. Our patient's thrombus was highly mobile but not freely floating.

Although the involved mechanisms have not been fully elucidated, patients infected with human immunodeficiency virus (HIV) have an increased risk of developing thrombosis. Thrombotic events may be as high as ten times more prevalent in this group as in the general population [5]. The increased tendency for thrombosis could be due to decreased natural anticoagulants such as protein C, S and antithrombin III; presence of antiphospholipid antibodies, endothelial and platelet activation; malignancy; systemic infections and lupus anticoagulant [5]. The use of highly active antiretroviral therapy is also associated with increased thrombosis due to increases in plasminogen activator inhibitor I (PAI-I), fibrinogen and lipid levels [6]. It is possible that our patient's thrombus embolised from the peripheral veins and stuck in the IVC and right atrium. It subsequently enlarged, calcified and most likely formed a nucleus for further thrombus deposition. Her HIV/AIDS status, the use of HAART, chronic kidney disease and cigarette smoking possibly favoured the development of a hypercoagulable state and thrombosis.

A transthoracic echocardiogram can be used to diagnose and provide information on the location, echogenicity and morphology of the thrombus [7]. A transoesophageal echocardiogram and cardiac computed tomography are used for additional description [7]. However, differentiating chronic calcified intracardiac thrombus from other causes of intracardiac calcification such as neoplasms may be difficult [2]. In our patient, the diagnosis was made after surgical excision.

Although there is no clear consensus of the preferred treatment option, recommended treatment options include surgery, thrombolysis and anticoagulation [8]. Nevertheless, any decision should be individualised taking into consideration the extent, size, shape, and mobility of the thrombus as well as pre-existing pulmonary embolism/deep vein thrombosis, and cardiopulmonary reserve [8]. Surgery was an option in in our patient because of the big size of the thrombus, its mobility and polypoid appearance. The mass had not regressed despite being on anticoagulation for over two years. The patient had worsening dyspnoea probably from multiple small pulmonary emboli.

## Conclusion

HIV/AIDS is associated with a prothrombotic state with a considerable risk of thromboembolism including intracardiac thrombus. Right atrial thrombi may result from peripheral venous thrombosis and are pointers of impending pulmonary embolism. This case illustrates a large calcified inferior vena caval and right atrial thrombus that probably migrated from peripheral veins. While the management of an intracardiac thrombus remains challenging, surgical removal of calcified thrombus may be an option especially in patients with low risk of surgical complications.
